# Microfluidic live‐cell imaging of *Aspergillus fumigatus* and *Candida albicans* hyphal growth treated with AmBisome and Caspofungin

**DOI:** 10.1111/jmi.70053

**Published:** 2025-12-18

**Authors:** D. D. Thomson, R. Inman, S. Nye, E. M. Bignell

**Affiliations:** ^1^ Medical Research Council Centre for Medical Mycology at the University of Exeter Department of Biosciences, Faculty of Health and Life Sciences Exeter UK; ^2^ Manchester Fungal Infection Group, Division of Infection, Immunity and Respiratory Medicine, Faculty of Biology, Medicine and Health University of Manchester Manchester UK; ^3^ Living Systems Institue Univeristy of Exeter Exeter UK

**Keywords:** fungal hyphae antifungal microfluidic yeast live‐cell

## Abstract

Hyphal forms of human pathogenic fungi cause invasive disease in humans, but the hyphal response to antifungals is understudied. In the major fungal pathogens *Aspergillus fumigatus* and *Candida albicans*, we used microfluidic‐coupled, fluorescence‐mediated live‐cell imaging to capture the real‐time responses of fungal hyphae to clinical concentrations of AmBisome or Caspofungin. In both fungi, AmBisome exposure caused rapid growth arrest (<15 min) and subcellular reorganisation and, in *C. albicans*, localised expansions of lipid‐like structures from the hyphal perimeter. Responses to Caspofungin exposure were slower, with initial lytic effects occurring after 1.5 or 4 h in *A. fumigatus* and *C. albicans* hyphae, respectively. While *C. albicans* hyphae undergo unsalvageable hyphal lysis in response to Caspofungin, *A. fumigatus* exhibits several compensatory growth behaviours, including a novel resuscitative growth response, that circumvents lytic events to maintain apical and sub‐apical hyphal growth. This study reveals how the differing biologies of the two pathogens affected outcomes and contributes to the highly disparate rates of antifungal efficacy amongst commonly used drugs, where spore/yeast‐derived inhibitory doses may be underestimated to arrest/kill the invasive hyphal morphotypes in vitro.

Human pathogenic cause >2 M deaths per year and we have a limited number of antifungals in the clinic to combat these infections. Those drugs are increasingly meeting resistance in killer fungi and our understanding of drug responses are limited. Our main assessment of antifungal resistance comes from end‐point 48 h drug culture of the fungus and is performed by eye for presence of absence of growth in a well. Further, these tests are performed on the pre‐invasive form of the fungus, the yeast or spore (for speed and simplicity), not the invasive filamentous form present during systemic infection of organs in humans.

This work shows how the invasive filamentous form of two major human fungal pathogens respond to two frontline clinical antifungal drugs with real time microscopy. We describe the cell death and/or adaptive growth responses via live‐cell microscopy to understand the morphological and cellular responses over time. We were able to do this by coupling fluorescently engineered pathogens and live‐cell 4D microscopy to microfluidic delivery of culture media and/or antifungal drug. We used the triggerable microfluidics to first establish invasive filamentous growth without drug, and keep the cells in the same focal plane (with shallow roof that kept the filaments growing up out of the focal plane). We then triggered the switch to media with drug(s) to perfuse drug while observing comparative cellular responses live, up to 10 h in two pathogenic fungi.

In both pathogens (*Aspergillus fumigatus* and *Candida albicans*) we saw similar immediate responses to one drug (Ambisome), which inhibited filamentous growth almost immediately. In stark contrast, Caspofungin induced different but continued forms of growth to known inhibitory doses against invasive fungal filaments. *A. fumigatus* (a filamentous mould fungus) appeared hard‐wired to continue filamentous growth by various compensatory regenerative growth forms, including a novel ‘resuscitative’ form we describe which occurs after the drug causes filament bursting. However, *C. albicans* filaments (a polymorphic yeast) respond by transitioning from filamentous to yeast growth (all filaments eventually burst, leaving only budding yeast). This study details the short‐ and long‐term responses of invasive pathogenic fungal filaments to drugs and highlights that the traditional spore/yeast‐derived inhibitory doses for these fungi may be insufficient for the invasive form of the fungus, where more attention to the filamentous form is needed.

## 1 INTRODUCTION

End‐point routine Antimicrobial Susceptibility Testing (AST) of yeast and spores is simple to implement and highly reproducible in the clinic to determine minimum inhibitory and effective concentrations (MICs and MECs, respectively) of antifungal drugs.^1^, ^2^ However, in two major pathogens *A. fumigatus* and *C. albicans*, the filamentous hyphal form is the main driver of invasive disease pathology,^3^ where antifungals will be targeted. A complementary approach is required to study the effect of drug exposure on the invasive hyphal form of pathogenic fungi, since it has been reported that differing morphologies of the same fungal isolate can exhibit markedly different MICs.^4^This, coupled with the fact that frequently used antifungals such as Caspofungin do not have established EUCAST‐breakpoints,^2^ highlights the need to understand the growth dynamics in major invasive fungal pathogens.

Longitudinal analyses of antifungal drug responses in fungi have focused largely upon the action of the cell wall‐active echinocandin, Caspofungin. Caspofungin induces hyper‐branching and tip lysis followed by de novo intra‐hyphal growth within the lysed hyphal tips when initially applied to *A. fumigatus* spores at clinically relevant doses,^5^, ^6^, ^7^ or hyphae at significantly higher^8^ concentrations. Similarly, *C. albicans* yeast cells subjected to half‐MIC Caspofungin on solid media also generated hyphae susceptible to tip lysis^9^ as well as exhibiting a modified cell wall ultrastructure and lipid localisation after MIC drug exposures.^10^, ^11^, ^12^, ^13^ These powerful studies have revealed intriguing cellular responses to the echinocandin drug class, identifying novel and shared antifungal resistance strategies amongst fungal pathogens, such as active cell wall remodelling and morphological aberrancies. Such studies highlight the dearth of information on the dynamic modes of action of other antifungal drugs. For example, despite decades of approved clinical use to treat fungal infections, the dynamic fungal cellular responses to liposomal Amphotericin B (AmBisome) have yet to be visualised over time. However, one study in *Saccharomyces cerevisiae* yeast has documented rapid depletion of ergosterol under Amphotericin B within <30 min, quickly followed by cell death.^14^ AmBisome is a lipid formulation of Amphotericin B, which specifically delivers Amphotericin B to fungi to extract ergosterol from the fungal cell membrane like a sponge, resulting in fungicidal activity against both yeast and hyphae.^14^, ^15^ Microfluidic live‐cell imaging (Mf‐LCI) of multiple experimental conditions under long‐term microfluidic perfusion is a powerful tool for observing and comparing real‐time responses of living fungal cells to antifungal drugs.^8^ Complementary to initial AST end‐point analyses, Mf‐LCI permits longitudinal and comparative analyses of single‐cell dynamic responses of invasive hyphae to perfused MIC drug exposures under the microscope. Furthermore, the in‐built flexibility of microfluidic drug delivery regimens permits real‐time analysis of fungal responses to introduction, withdrawal and reintroduction of antifungal drugs as well as user‐determined emphasis on specific fungal morphotypes. We applied Mf‐LCI to perform a comparative analysis of synchronously captured responses of living *A. fumigatus* and *C. albicans* hyphae, to Caspofungin and AmBisome exposure. This study describes the observed distinct and shared cellular adaptations, occurring over differing timescales, in two major human fungal pathogens in response to clinical antifungals.

## 2 RESULTS AND DISCUSSION

**FIGURE 1:**
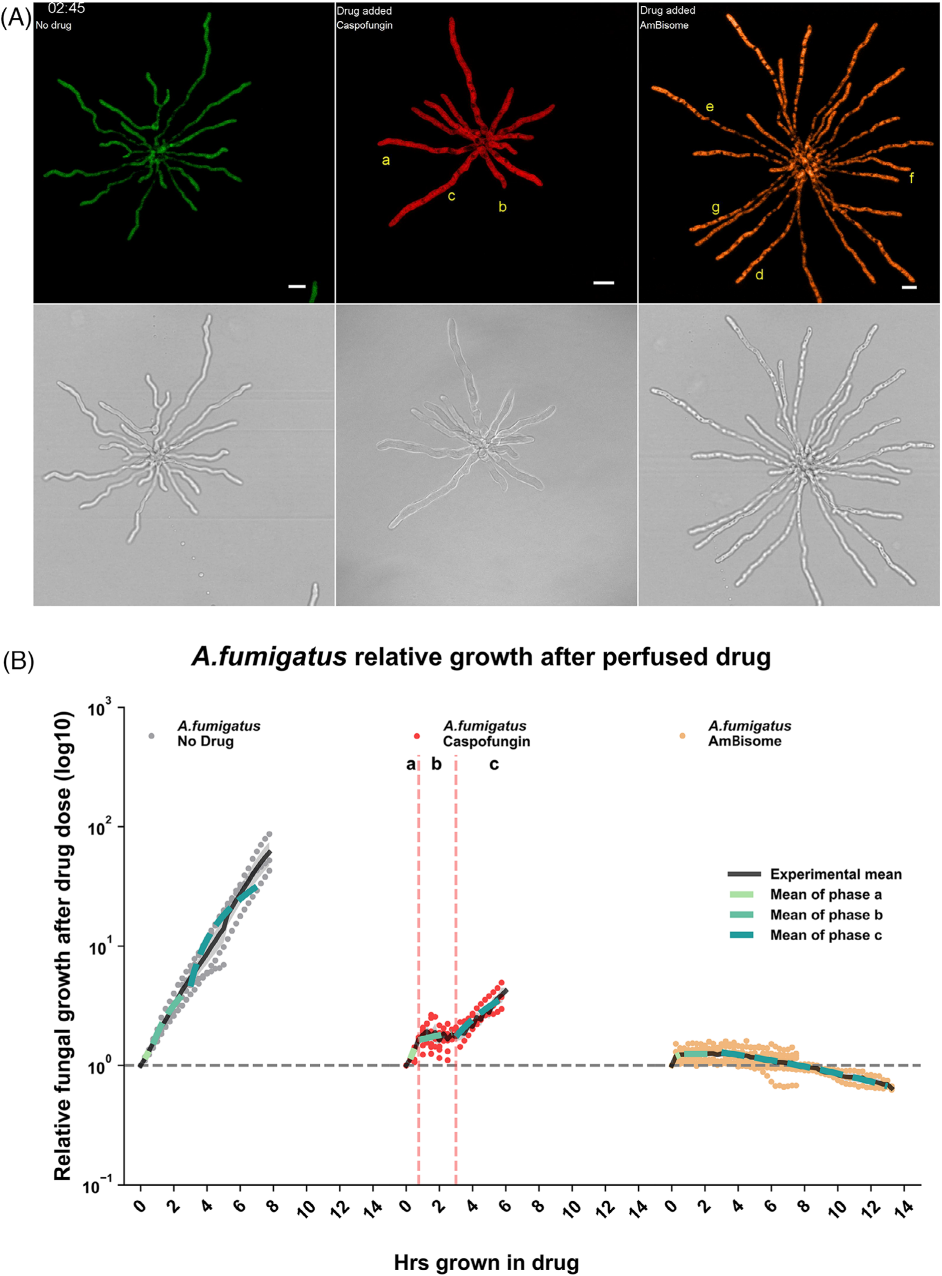
Hyphal growth dynamics of *A.fumigatus* after Caspofungin and AmBisome perfusion. (A) Montage from exemplar Movie 1 of fluorescent *A. fumigatus* responding to 0.5 µg/mL Caspofungin and 0.125 µg/mL AmBisome. Untreated (green; left column), Caspofungin (red; middle column) and AmBisome (amber; right column) treated *A. fumigatus* hyphae growing in a Cellasic microfluidic chamber. After growth in media without drug, new media without drug (green) or drug (red/amber) were perfused into the chamber. The fluorescence images were pseudo coloured in the top row, while the bottom row displays the transmitted light images. Hyphal events are denoted with the following letters to indicate key events occurring in Movie 1: (a) Caspofungin‐treated hyphal tips lyse, maintain an Apical Post‐lytic Extracellular fluorescent Signal (APES) and resuscitate. Subapical branching is also evident in the compartment behind the apex. (b) Caspofungin‐treated hyphal tips lyse, maintain a fluorescent apical tip and do not resuscitate. Subapical branching is evident in the basal compartment. (c) Caspofungin‐treated hyphal tips lyse and undergo intra‐hyphal growth at the nearest apical septa. The subapical compartment also undergoes resuscitative growth with septum formation. The basal compartment undergoes retrograde intra‐hyphal growth from the basal branch septum toward the mother spore body. (d–g) AmBisome‐treated hyphae undergo presumed vacuolation followed by: (d) maintenance of vacuoles, (e) vacuole lysis and diminishing cytosolic fluorescence protein, (f) sudden hyphal lysis, and (g) vacuole lysis with no diminishing of cytosolic fluorescence protein. Scale bar = 10 μ m. (**B)** Image‐based fungal growth dynamics to antifungal agents, based on segmented fluorescent cytosol from time‐lapse images. *A.fumigatus* relative growth after drug perfusion was plotted. Each data point is expressed as relative growth after drug perfusion. Black solid lines represent experimental mean fit of all the technical replicates (No drug = 4, Caspofungin = 10 and AmBisome = 8) from at least three biological replicates. Additionally, three distinct phases of growth are marked by notations of ‘a’, ‘b’ and ‘c’ and the vertical dashed lines in the Caspofungin treatment. The mean growth rate of these phases are additionally mapped onto the other conditions as dashed lines by the colour code indicated in the figure legend. The standard error of the experimental mean plots are indicated by shaded areas around the curve. Significance testing was performed using a One‐way ANOVA test between the same phase of growth across conditions (comparing no drug, Caspofungin, AmBisome); phase a *p* < 0.001, phase b *p* < 0.001 and phase c = 0.002. A further One‐way ANOVA test was used between the same condition across different phases of growth (comparing phases a–c); No drug *p* < 0.001, Caspofungin *p* <0.001 and AmBisome *p* = 0.284. Statistical testing between specific conditions and phases of growth were performed with a Dunnet's multiple comparisons analysis which are summarised in Table S2.

In order to study clinically relevant in vitro responses of invasive fungi, the EUCAST‐guided^1^, ^2^ inhibitory drug concentrations were determined for each pathogen with Caspofungin and AmBisome (Table S1). These clinical‐standard inhibitory doses and culture conditions were used to track the immediate to medium‐term responses of growing fungal hyphae to antifungal drug exposure in our perfused Mf‐LCI set up. The Cellasic microfluidic plates used did not significantly sequester the drug, suggesting that the inhibitory effect for both drugs remained stable following perfusion File (S1). We coupled Mf‐LCI with fungal strains expressing genetically encoded fluorescent cytosol reporters to visualise and quantify fungal biomass, cellular integrity (lysis or leakage) and changes to sub‐cellular organisation. Although the effect of shear stress was not measured or accounted for in these experiments, we observed no directional effects on hyphal growth responses by the uni‐directional flow of media and drug into the Cellasic microfluidic chamber. To track and quantify fungal biomass (2D cross‐sectional area) from our 4D fluorescence image data, in response to drug perfusion, we applied 4D image processing to Mf‐LCI data over multiple biological replicates in the presence or absence of drug for both pathogens (Figure S2). The growth data was then expressed as relative growth upon drug perfusion. In order to compare the growth phases of each condition, phases of growth were determined from the non‐linear Caspofungin relative growth curves in each pathogen which approximated early‐ (a), mid‐ (b), and longer‐term phases (c; Figures 1B and 3B). These phases were determined by identifying maxima and minima in the rate of change (ROC) of relative growth. As the maxima and minima are indicative of fastest and slowest growth, the moments of change of distinct growth phases were determined as the time points where the ROC curve is at half‐maximum value (Figure S1).

### 2.1 A. fumigatus antifungal responses

**FIGURE 2:**
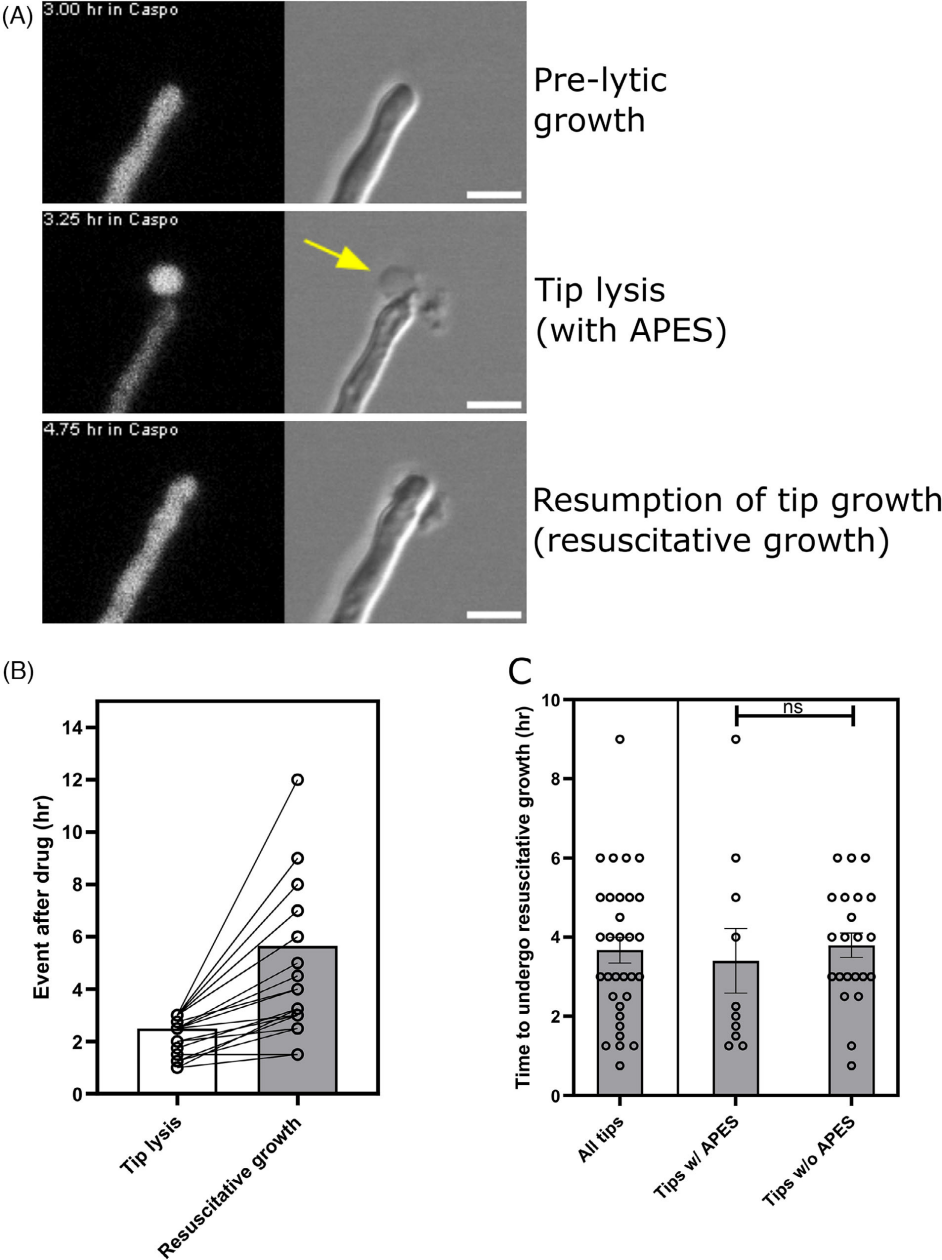
Resuscitative growth in *A. fumigatus* after Caspofungin perfusion. (A) Representative timelapse images of *A .fumigatus* resuscitative fungal growth after microfluidic perfusion of Caspofungin illustrating analysed events of MEC Caspofungin treatment. Left panels are YFP (cytosolic) fluorescence images and DIC images are on the right panels. Presence of an Apical Post‐lytic Extracellular fluorescent Signal (APES) is marked by the yellow arrow. Scale bar = 5 µm. (**B)** Analysis of resuscitative events after drug perfusion. Timing of hyphal tip lysis and their subsequent tip regrowth via resuscitation, was tracked in individual hyphae from live‐cell movies containing *n* = 33 resuscitative hyphae from 4 biological replicates. The time lag between tip lysis and resuscitative regrowth events for single hyphae are linked by a connecting line for those 33 hyphae. In each column, the bar is the mean time for each event described. (**C)** Analysis of resuscitative growth onset after initial tip lysis with, or without, an APES. The delay between initial apical tip lysis and the onset of resuscitative growth is plotted for all resuscitative hyphae. Of all the resuscitated tips, those which did (*n* = 10) or did not (*n* = 22) have an APES are plotted. For each condition, individual hyphal replicates from 4 biological replicates are illustrated by open circles, where the mean value is illustrated as a box and the error is the SEM. Comparisons were not significant (*p* = 0.66) using a two‐tailed unpaired Student's *t*‐test with Welch's correction.

Upon perfusion with 0.5 µg/mL (MEC) Caspofungin, pre‐germinated *A. fumigatus* hyphae continue to grow at the same rate as the no drug condition until the onset of apical lysis (Figure 1B, phase a; *p* = 0.244), which occurred at mean time of 2.5 h after drug (Figure 2B).

Typical apical lysis is illustrated at 75 min post‐exposure in exemplar Movie 1 (Movie 1 hyphae a and b 03:15). The remaining hyphae in exemplar Movie 1 lysed within 105 min of each other (75–180 min). Upon the onset of tip lysis, fungal growth was significantly reduced compared to growth in the absence of drug over the same phase of growth (Figure 1B, phase b; *p* < 0.001). As previously described,^7^, ^8^ frequent and heterogeneous hyphal outcomes were observed, including apical and subapical compartment lysis (Movie 1 hypha c 03:45 and 05:00), subapical branching (Movie 1 hypha c 04:00–08:00) and de novo intra‐hyphal growth (Movie 1 hypha c apex 04:45–08:00). Intrahyphal apical growth of a new hyphal tip originates from the apical septum and continues within the lysed apical compartment, past the site of previous tip lysis.^7^, ^8^ In contrast, we additionally observed an example of retrograde intra‐hyphal growth upon lysis of a basal non‐apical hyphal compartment. Here, de novo intra‐hyphal growth occurred from the local branch‐septum back into the lysed basal compartment containing the mother spore (Movie 1 hypha c spore compartment; Movie S1). Immature germlings lacking septa (i.e. non‐compartmentalised) were unable to display any form of residual growth once lysed and no longer exhibited fluorescence, indicating a complete loss of metabolic activity after 6 h under Caspofungin perfusion (data not shown^16^).

We observed a new distinct type of regenerative growth behaviour in 40% (33/81) of lysed hyphal tips that were tracked after a lytic event (designated here as resuscitative growth), involving full recovery of intracellular cytosolic fluorescence and growth capacity; without the need for de novo hyphal growth that typifies the intra‐hyphal growth response.^7^ Resuscitated hyphae took between 45 min Movie (S2; hypha a) and 9 h (Figure 2C) to presumably localise cell wall repair mechanisms, recover cytosol, turgor and establish the resumption of polarised tip growth. Resuscitated growing tips were still susceptible to subsequent Caspofungin‐induced lytic activity. Amongst an exemplar population of 12 lysing hyphal tips in Movie 1, 4 hyphae exhibited an Apical Post‐lytic Extracellular fluorescent Signal (APES). Three of these hyphae underwent the resuscitative hyphal growth programme involving replenishment of fluorescence and turgor within the lysed compartment (Movie 1 hypha a). A fourth lysed hypha exhibiting the post‐lytic fluorescence signal did not resuscitate but instead continued growth by branching from its basal septa, out‐with the lysed apical compartment (Movie 1 hypha b; Movie S1 hypha b). In all resuscitative hyphal tip events captured in this study, 30% (10/33) were associated with highly localised APES at the apex of lysed hyphae (Movie 1 hypha a; Movie S3). However, the presence of this APES structure did not appear to have an impact on hyphal resuscitation, where the time for hyphae to resuscitate and continue growth was not impacted (Figure 2C; *p* = 0.667), compared to resuscitative hyphae lacking this APES structure Movie (S2; hypha a). However, we noted that the slowest hypha to resuscitate amongst each biological replicate tended to have APES associated. Subapical hyphal compartments also illustrated post‐lytic resuscitative growth (Movie 1 hypha c 03:45) which were also still capable of co‐ordinating septum formation (2 h later), indicating it is not a phenomenon exclusive to hyphal tip recovery. The regenerative post‐lytic behaviours described here lead to a recovery in relative growth rate (phase c; 0.69) comparable to the initial pre‐lytic growth phase (phase a; 1; Figure 1B, phase a–c; *p* = 0.058), but was still significantly slower compared to no drug in the same growth phase (Figure 1B, phase c; 6.74; *p* <0.003). The post‐lytic cellular responses we map here in time, occurring at MEC drug concentrations, may contribute to the failure of MEC Caspofungin to sterilise *A. fumigatus* hyphae after 48 h in vitro^7^ and possibly in vivo.

In stark contrast to Caspofungin, *A. fumigatus* hyphae perfused in the same manner with 0.125 µg/mL AmBisome elicited an immediate homogenous cessation of growth (<15 min; Movie 1 far right), which did not recover after 6 h perfusion (Figure 1B; *p* = 0.284). This rapid response to MIC AmBisome was associated with subsequent rapid changes in the subcellular ultrastructure based on the cytosolic fluorescence (Movie 1 far right). Ultrastructure changes included the appearance of cytosolic fluorescence occlusions that co‐localised with regions in DIC images of different refractive indices to cytosol, which resemble vacuoles. However, without probes such as FM4‐64 (N‐[triethylammoniumpropyl]‐4‐[p‐diethylaminophenylhexatrienyl] pyridium dibromide) or CMAC (7‐amino‐4‐chloromethylcoumarin),^17^ we cannot definitively identify these structures as vacuoles. The peak number of non‐fluorescent cytosolic occlusions were formed in hyphae after approximately 45 min of AmBisome exposure (Movie 1 far right).

As growth was completely inhibited by MIC AmBisome and subcellular reorganisation progressed, amongst a typical exemplar population of 24 fungal hyphae in Movie 1, three subsequent responses were observed: 11/24 hyphae maintained their presumed vacuoles (46%; Movie 1; hypha 1d), 8/24 hyphae underwent presumed vacuole lysis after 1 h 45 min exposure (33%; Movie 1; hypha e) and 5/24 hyphae underwent cellular lysis after 4 h 15 min exposure (21%; Movie 1; hypha f). Presumed vacuole lysis was followed by extinction of cytosolic fluorescent signal in one hypha within the subsequent 2 h (Movie 1; hypha e; 05:30‐07:30) suggesting a possible leaching of cytosol, since photobleaching was not observed in the other hyphae within the studied timeframe (Movie 1; site g). The relative rates of growth of AmBisome treated hyphae were negligible and trending negatively, presumably due to the cidal observations described above (Figure 1B; phase a–c; 0.16 to –0.06).

### 2.2 C. albicans antifungal responses

**FIGURE 3:**
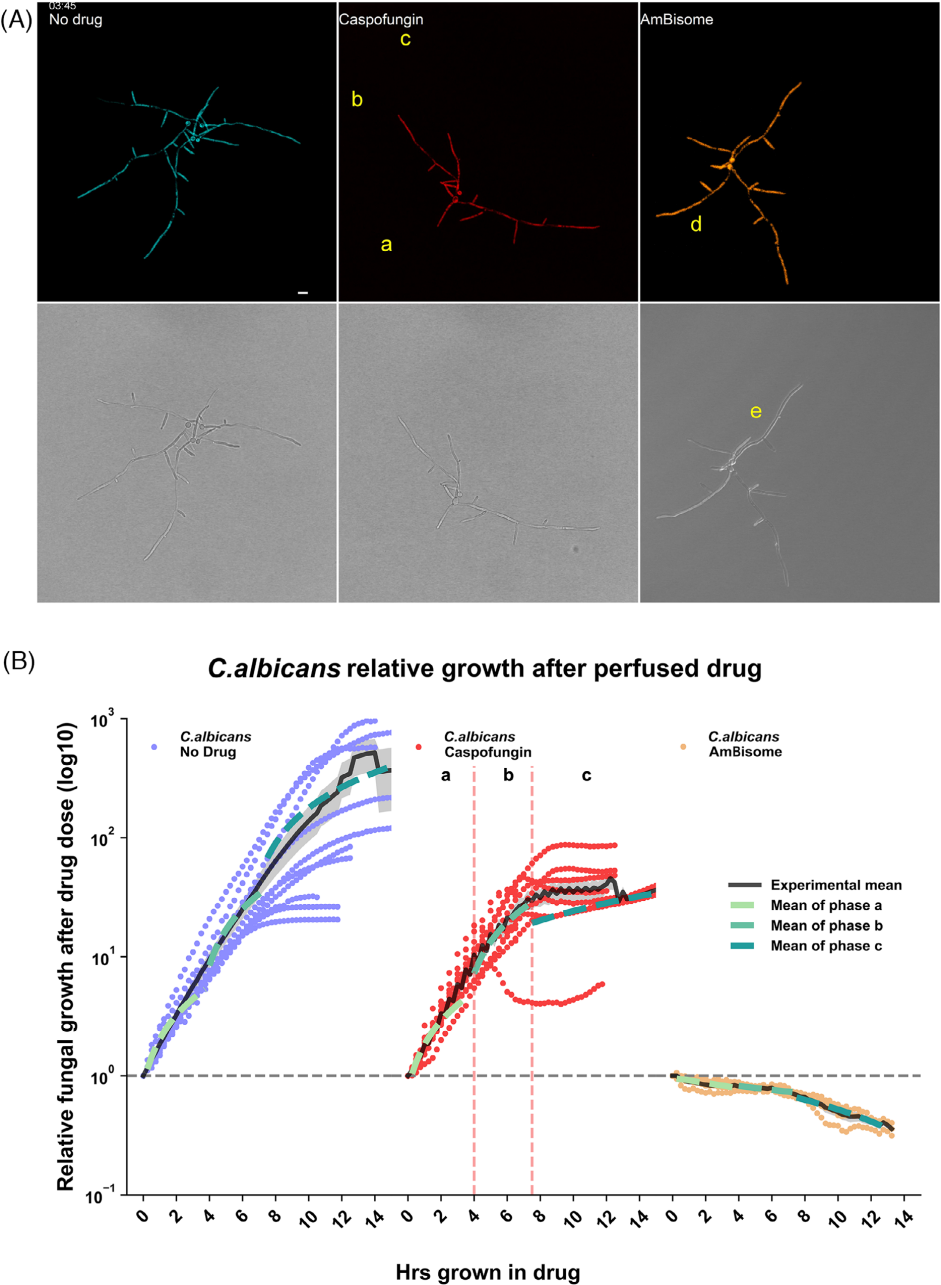
Hyphal growth dynamics of *C. albicans* after Caspofungin and AmBisome perfusion. (A) Montage from Movie 2 of synchronised movies of fluorescent *C. albicans* responding to 0.08 µg/mL Caspofungin and 0.5 µg/mL AmBisome. Untreated (cyan; left), Caspofungin (red; middle) and AmBisome (amber; right) treated *C. albicans* hyphae growing in a Cellasic microfluidic chamber. After growth in media without drug, new media without drug (green) or drug (red/amber) are perfused into the chamber. The fluorescence images were pseudo coloured in the top row, while the bottom row displays the transmitted light images. Hyphae denoted with the following letters indicate key events occurring in the movies: (a) Caspofungin‐treated hyphae begin lysing. (b) Caspofungin‐treated hyphae begin branching, become less polarised and revert to budding growth. (c) Caspofungin‐treated hyphal tips begin septating and reverting to budding growth. (d) AmBisome‐treated hypha undergoes presumed vacuolation followed by vacuole lysis and a diminishing of fluorescence protein. (e) AmBisome‐treated hypha undergoes development of lipid‐like protrusions from fungal cell periphery. Scale bar = 10 μ m. (B) Image‐based fungal growth dynamics to antifungal agents, based on segmented fluorescent cytosol from time‐lapse images. *C. albicans* relative growth after drug perfusion was plotted. Each data point is expressed as relative growth after drug perfusion. Black solid lines represent experimental mean fit of all the technical replicates (No drug = 10, Caspofungin = 12 and AmBisome = 4) from at least three biological replicates. Additionally, three distinct phases of growth are marked by notations of ‘a’, ‘b’ and ‘c’ and the vertical dashed lines in the Caspofungin treatment. The mean growth rate of these phases are additionally mapped onto the other conditions as dashed lines by the colour code indicated in the figure legend. The standard error of the experimental mean plots are indicated by shaded areas around the curve. Significance testing was performed using a One‐way ANOVA test between the same phase of growth across conditions (comparing no drug, Caspofungin, AmBisome); phase a *p* < 0.001, phase b *p* = 0.026 and phase c = 0.011. A further One‐way ANOVA test was used between the same condition across different phases of growth (comparing phases a–c); No drug *p* < 0.001, Caspofungin *p* = 0.002 and AmBisome *p* = 0.288. Statistical testing between specific conditions and phases of growth were performed with a Dunnet's multiple comparisons analysis which are summarised in Table S2.

**FIGURE 4:**
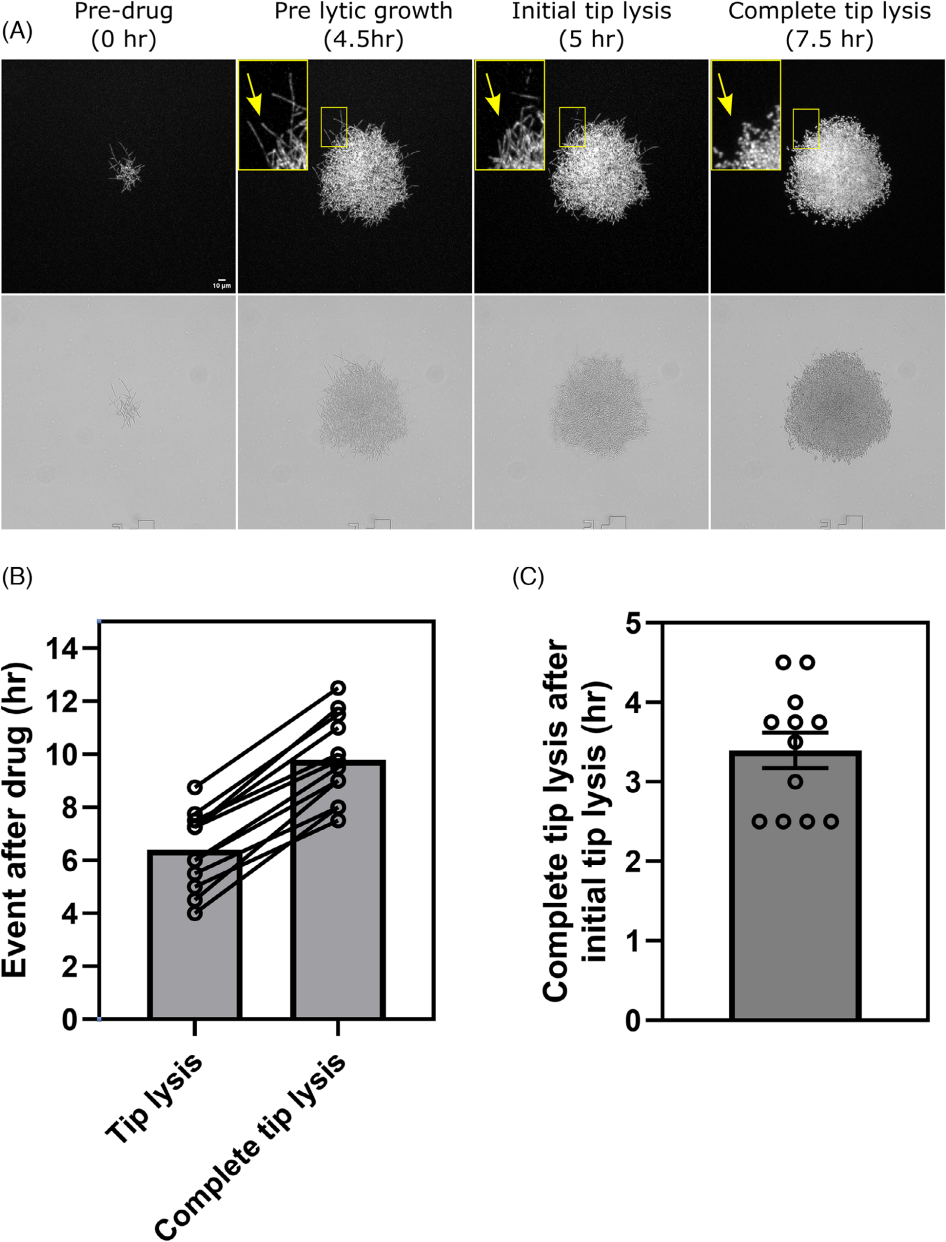
*C. albicans* hyphal tip lysis events after Caspofungin perfusion. (**A)** Representative timelapse images of *C. albicans* fungal growth after microfluidic perfusion of Caspofungin illustrating analysed events of MIC Caspofungin treatment. Top panels are ACT1‐GFP (cytosolic) fluorescence images and transmitted light images in the lower panels. Scale bar is indicated. Zoomed fluorescence inset images show individual hyphae undergoing initial tip lysis marked by the yellow arrow. (**B)** Analysis of live‐cell imaging data sets of *C. albicans* hyphal tips undergoing initial en‐mass hyphal tip lysis and the subsequent completed hyphal lysis after Caspofungin perfusion for each analysed data set. Individual live‐cell data sets are illustrated by open circles and the occurrence of initial tip lysis and subsequent total tip lysis from the same data set is linked with a line between the events, where the mean time for each event to occur is illustrated as a box. (**C)** The time taken for *C. albicans* hyphae to completely lyse after the initial hyphal lysis event. *N* = 12 live‐cell hyphal data sets from 3 biological replicates. For each condition, individual live‐cell data sets are illustrated by open circles, where the mean value is illustrated as a box and the SEM is displayed.

Similar to observations of *A. fumigatus*, we found that exposure of *C. albicans* hyphae to MIC Caspofungin (0.08 µg/mL) did not completely inhibit growth after 6 h of perfusion (Movie 2). Initially, *C. albicans* hyphae grew at the same relative growth rate as untreated hyphae (1.06 vs. 1.19; Figure 3B phase a; *p* = 0.706). Hyphal growth continued growing and did not begin lysing until 4 ‐ 8.75 h of perfusion in the Mf‐LCI platform (Figures 3 and 4B; Movie 2; site a; 08:15), which resulted in a non‐significant 27% drop in relative growth rate (6.11), when compared to untreated hyphae (8.39; Figure 3B phase b; *p* = 0.524). Interestingly, although slower than the no drug condition, the mean relative growth rate of phase b *C. albicans* hyphae in Caspofungin (6.11) was almost six times faster than the initial phase a growth (1.06; Figure 3B phase b; *p* = 0.003), possibly represented by the heterogeneous nature of when tip lysis begins to occur within that growth phase (Figure 4B).

Unlike *A. fumigatus*, *C. albicans* hyphae do not exhibit apical filamentous salvage growth behaviours following tip lysis. Instead, en‐mass hyphal tip lysis (Figure 4) was consistently followed by a switch to isotropic budding growth in the remaining viable elements of the micro colonies (Movie 2; 08:00–10:00). A longer‐term population‐switch to budding growth at branch sites (Movie 2; site b; from 07:15) and leading hyphal tips (Movie 2; site c; 07:45–10:00) then dominated the growth response. The timing of initial tip lysis and the moment of complete tip lysis was analysed, where it took between 2.5 and 4.5 h (mean 3.4 h) after initial tip lysis for yeast to dominate the remaining population of viable fluorescent cells (Figure 4C). The density of some *C. albicans* microcolonies meant that only the peripheral individual hyphae were analysed, where the core was too dense to identify single cells (Figure 4A). Further, budding growth was maintained and somewhat protected from lysis in the dense central mass Movie (S4), possibly representing a drug penetration defect in MIC Caspofungin (File S1), which may impact the longevity of yeast‐like products in our system. Newly formed isotropic cells were capable of continued budding growth, but were still susceptible to Caspfungin‐induced lysis. *C. albicans* yeast, derived from lysed hyphae, can maintain a steady‐state level of residual growth during phase c (1.42) which is comparable to the pre‐lytic phase a (1.06; Figure 3B; *p* = 0.952). Further, we found that reintroduction of drug‐free RPMI‐MOPS media after 6 h of Caspofungin exposure at 37°C resulted in no recovery of filamentous growth. Instead, further steady‐state budding growth and lysis of fluorescent yeast cells continued for 5 h in the absence of drug Movie (S5).

Similar to *A. fumigatus*, in response to AmBisome MIC treatment (Movie 2), *C. albicans* hyphae were observed to immediately form cytosolic fluorescent occlusions (inferred as vacuoles; described above) in <15 min and did not grow between phase a–c (Figure 3B; *p* = 0.288). MIC AmBisome induced a negative rate of relative growth after perfusion (Figure 3B phase a; ‐0.04). The dynamic fluorescence occlusion structures subsequently lyse (Movie 2; beginning at 08:15), leading to diminishing cytosol fluorescence in hyphae (Movie 2; site d), possibly due to the fungal membrane becoming porous via AmBisomes’ action on membrane ergosterol.^14^, ^15^ This was coupled with maintained negative relative growth rates throughout (–0.05; Figure 3B phase c; *p* = 0.750) as the cidal activity continued, leaving less fungal fluorescent cytosol over time. Suspected extracellular fungal membrane detachment was also observed under carefully tuned differential interference contrast (DIC) optics, where we visualised refractile lipid‐like protrusions originating along the hyphal perimeter after 2 h 15 min of perfusion with MIC AmBisome (Movie 2e). These refractile structures were more pronounced and balloon‐like after 45 min of perfusion with higher 4 µg/mL doses of AmBisome Movie (S6). Only AmBisome‐treated *C. albiacans* hyphae displayed this phenotype. Growth arrest in AmBisome‐treated *C. albicans* cells was similar to *A. fumigatus* in a highly homogenous manner and time‐frame; this may be due to the ubiquitous non‐localised distribution of the drug target (ergosterol) along fungal hyphae. The en‐mass subcellular re‐organisation in hyphae presumably reflects attempts to sequester imported Amphotericin B, as seen in *C. albicans* studies of drug uptake into vacuoles using fluorescence lifetime imaging,^18^ and may be associated with an active reduction in cytosolic volume or an arrest in the G1 cell cycle.^19^, ^20^

## 3 CONCLUSIONS

In this study we undertook a comparative live‐cell microscopy analysis of the dynamic hyphal responses to microfluidic Caspofungin and AmBisome exposure. We address how hyphae, rather than the more‐often studied yeast or spore form, respond to continuous perfusion of antifungal drugs. Additionally, via modified MIC testing, higher concentrations of both Caspofungin and AmBisome were required to inhibit growth of *A. fumigatus* germlings, compared to spores (Table S1), a phenomenon previously described for AmBisome.^4^ These data support that it is important to study the invasive morphotypes of invasive fungal pathogens, rather than the well‐studied yeast and spore forms.

Mf‐LCI of hyphae demonstrates in vitro that, under continuous Caspofungin perfusion, hyphae of the filamentous mould *A. fumigatus* remain viable and maintain hyphal growth via distinct modes (branching, hyper septation, anterograde/retrograde intra‐hyphal growth and the newly described resuscitative growth). A new phenomenon of residual post‐lytic apical cytosolic aggregates (the underlying basis of which is not currently understood) were identified to associate with a third of hyphal resuscitation events, possibly via the localised repair of ruptured cell wall and recovery of turgor.

Despite the ability to rejuvenate via such modes of growth, these hyphae remained susceptible to Caspofungin‐induced lysis. In the time‐frame analysed, fungal biomass relative to untreated hyphae was drastically reduced 10–20 fold. In contrast to the delayed growth inhibition to Caspofungin, AmBisome stopped all growth within 15 min exposure and only lost biomass over time, via loss of cytosolic fluorescent protein from lysed or possibly porous cells.

In response to MIC Caspofungin, the hyphae of the polymorphic fungal pathogen *C. albicans* take on average 2.7× longer to lyse (6.8 h) compared to MEC‐treated *A.fumigatus* hyphae (2.5 h) and switch to budding yeast‐form growth, with varying dynamics that may be dependent upon colony size. The *C. albicans* switch to isotropic growth under Caspofungin may have clinical implications for biofilm integrity and/or systemic dissemination in the blood stream. *C. albicans* similarly stopped growing and underwent suspected vacuolation and fluorescence cytosol loss in response to MIC AmBisome.

This study describes real‐time responses of fungal hyphae to antifungal drugs, documenting the occurrence and dynamics of highly prevalent fungal lysis and re‐growth events in vitro. It will be important to understand these events in a clinical context, where the immune system may be engaged by lysed and/or polymorphic fungal material evoked by antifungal treatment regimens. These responses may contribute to emergence of echinocandin resistance seen in the clinic and better‐inform future combinatorial treatment regimens to abate echinocandin‐driven resurgent growth. This study also clarifies the real‐time hyphal responses to MIC AmBisome in two major fungal pathogens.

## 4 METHODS

### 4.1 Fungal strains, MIC testing and culture conditions

*A. fumigatus* ATCC 46645 expressing cytosolic YFP [PgpdA::yfp(ptrA)] spores were streaked on Potato Dextrose Agar plates, cultured for 48 h at 37°C and harvested in water via Miracloth filtration, prior to Mf‐LCI. The yeGFP *C. albicans* strain (CAI4) *RPS1/RPS1*::*pACT1‐GFP* ^21^ was a gift from Alistair Brown (University of Exeter), streaked on Yeast Peptone Dextrose (YPD) agar plates at 30°C for 48 h. Colonies of *C. albicans* yeGFP yeast were picked from YPD agar plates and inoculated into YPD liquid culture for overnight incubation at 30°C and diluted in RPMI‐MOPS prior to Mf‐LCI.

RPMI‐MOPS (pH 7) fungal growth media were prepared by following the EUCAST guidelines.^1^, ^2^ Working concentrations of AmBisome were prepared for each experiment by adding 12 mL dd H_2_O to a vial of AmBisome powder to generate a vial of 4 mg/mL. This was used to generate a final concentration of Amphotericin B (within the AmBisome formulation) in RPMI‐MOPS as indicated in the results text. Stock Caspofungin was prepared to 10 mg/mL in DMSO. Working concentrations of Caspofungin were prepared in dd H_2_O for final dilution in RPMI‐MOPS as indicated in the text. Hyphal MIC testing of *A. fumigatus* was performed by germinating 1–2.5 × 10^5^ spores/mL in 100 µL RPMI‐MOPS (pH 7) at room temperature overnight in a flat‐bottomed 96 well plate, followed by an additional 8 h incubation at 37°C, until approximately 95 % of the spores had germinated and contained 1 septa. The plates were sealed with breath‐easy sealing membrane to prevent evaporation. This was followed by the addition of 100 µL of RPMI‐MOPS or RPMI‐MOPS containing double strength antifungal to each well. Growth inhibition was then recorded by visual inspection after 24 and 48 h incubation at 37°C.

### 4.2 Microfluidic live‐cell imaging

Microfluidic Cellasic plates were aspirated and perfused with RPMI‐MOPS in all wells to expel the plate antibiotic, in the CellASIC ONIX2 Microfluidic System (CAX2‐S0000; Merck, UK). Fluorescent fungal cells were diluted to a concentration of 1 × 10^5^ cells/mL in RPMI‐MOPS, where 200 µL of suspension was loaded into a YO4E Cellasic plate (Merck, UK). Fungal cells were perfused into the plate imaging chambers via microfluidic bursts at a pressure of 8 PSI for 5 s using the ONIX2 software (v 5.0; Merck, UK). This was repeated until a satisfactory cell distribution was achieved for good visualisation of subsequent single‐hypha growth without over‐crowding. Fungal spores/yeast were left to adhere for 30 min^22^ without flow, whereby RPMI‐MOPS was subsequently flowed into the chamber at 2 PSI continually to remove unadhered cells. Adhered cells were continually perfused with fresh RPMI‐MOPS until hyphae were established and ready for pre‐ and perfused‐drug imaging. *A. fumigatus* spores were pre‐germinated in the plate for 8–10 h at 37°C in RPMI‐MOPS at 1 PSI flow, prior to live‐cell imaging. *C. albicans* yeast were germinated in the microfluidic plate at 37°C for 2–4 h in RPMI‐MOPS at 1 PSI flow, prior to live cell imaging. The plate was mounted on the microscope and a solution switching program was established via the ONIX Cellasic controller and software. A continual flow of fresh RPMI‐MOPS media was set for 2–6 h at 1 PSI, depending on the size of the microcolony, then followed by freshly perfused AmBisome or Caspofungin‐containing RPMI‐MOPS at initially 4 PSI for 5 min, then 1 PSI continuously, up to 14 h.

### 4.3 Drug efficacy testing after Cellasic plate perfusion

Drug efficacy in the Cellasic microfluidic YO4E plates were tested by loading the effective concentrations of drug into the plate (wells 1–6) and flowing them through the plate without cells to the waste wells (7 and 8) at 37°C for 3 h at 8 psi flow rate. The effluent was collected from the waste well and used to perform MIC testing following EUCAST guidelines described earlier on *A.fumigatus* spores and *C. albicans* yeast. Their inhibitory effect was compared with the same concentration of drug that had not been flown through the Cellasic plate. Growth inhibition of *A.fumigatus* and *C. albicans* was measured by optical density (OD) at OD600 nm and OD530 nm, respectively, on a Tecan Spark OD plate reader after 24 h growth at 37°C.

### 4.4 Image acquisition and movie production

Under microfluidic perfusion, 3D confocal and widefield image stacks (7 slices, 3 µm apart) were acquired every 15 min on a 37°C temperature controlled (Cube and Box, CH) Leica SP8× confocal microscope (Leica Microsystems, Germany) equipped with a dry Leica 40×/0.85NA HCX PL APO CS, or, when required for the visualisation of fungal refractile lipid‐like protrusions, an oil immersion 63×/1.4NA HC PL APO CS2 objective lens with tuneable DIC optics. Fluorescence images of yeGFP *C. albicans* and eYFP *A. fumigatus* strains were acquired with 488 nm and 514 nm argon lasers, respectively, with appropriate HyD point detection windows in LASX software (v.5.1.0 Leica Microsystems, Germany). The laser intensity and exposure to the cells were kept to a minimum (<1%) to avoid photobleaching and cellular phototoxicity. Associated transmitted images were acquired with a transmitted light detector. Additional widefield acquisitions were performed on a temperature regulated Zeiss AxioObserver Z1 equipped with a Hamamatsu Fusion sCMOS camera and a 20×/0.8NA Plan‐apo lens with a GFP filter set (Zeiss #62) which captured both GFP and YFP expressing fungal strains. Multiple fields of view/volumes were captured per biological replicate. Fluorescence 4D image data sets were imported as technical replicates (from at least 3 biological repeats) into FIJI^23^ and 3D‐projected by standard deviation. Time‐series images were then registered in FIJI using StackReg^24^ to eliminate spatial drift. Illustrative movie montages of synchronised capture and annotations of different conditions were created with the *Combine*, *Time stamper* and *label* features in FIJI.

### 4.5 Image segmentation and analysis of fungal growth

The cross‐sectional total area of fungal cytosol in a field of view, of each frame was segmented and quantified from fluorescence time‐series images in FIJI, for each culture condition. Briefly, each image was background subtracted (rolling ball 20 pixel radius) and blurred with a Median filter (radius 2 pixels) prior to applying a Percentile threshold and watershed of the fluorescent fungal channel segmentations. The total segmented cross‐sectional areas of fluorescent fungal mass per time point of the movies were then analysed using the *Particle Analyser* function in FIJI to measure the total cross‐sectional area of segmented fungal biomass at each time point. Total 2D cross‐sectional areas of fluorescent fungal cytosol per timepoint after drug addition were then transformed into relative growth upon drug perfusion (i.e. how much growth had occurred after drug was added). For the no‐drug controls, the same time point of drug entry from the neighbouring drug condition chamber of the Cellasic microfluidic plate was taken as the initial relative mass to report subsequent no‐drug growth.

### 4.6 Growth curve fitting and statistical testing

We used the stark Caspofungin response curve to map early (a), mid (b) and late (c) phases of growth onto the other AmBisome and no drug conditions for each pathogen. To identify these distinct phases of growth from the Caspofungin‐treated data for each pathogen, the relative growth data was imported into Python and plotted using the Matplotlib library.^22^ ROC of relative growth was calculated as the change between the previous and current growth value at each time point. The ROC data was smoothed using a Gaussian filter. Minima and maxima and the signal half‐maximum points were identified using Python library SciPy.^25^ To evaluate growth rates per replicate, a line (*y = mx+c*) was fitted to the identified time points (growth phases) of the relative growth curve. The lines were fitted using SciPy optimize.curve_fit, which uses non‐linear least squares to fit a function to data.^26^ Growth rates between drug treatments condition and between each growth phase (a–c) were initially assessed using one‐way ANOVA test. Post hoc comparisons, regardless of ANOVA significance, were performed using Dunnett's *t*‐test, with a Bonferroni‐adjusted significance threshold of *p* < 0.0083 to account for multiple comparisons (Table S2).

### 4.7 Analysis of morphological events after drug perfusion

Events such as individual hyphal tip lysis and the onset of resuscitative growth in *A.fumigatus* were manually scored in the timelapse movies, relative to the onset of drug perfusion. For *C. albicans*, due to the speed and density of its hyphal growth pattern, the moment of initial tip lysis and total tip lysis in the peripheral population (illustrated in Figure 4A) was recorded amongst hyphae after drug perfusion from the timelapse movies. These events were recorded in GraphPad Prism v 10.6.1, where graphs were also made. A Levene's test and a subsequent two‐tailed unpaired Student's *t*‐test with Welch's correction was also performed in Prism to compare the onset of resuscitative growth in tips associated with APES.

## DISCLAIMER

Movies 1 and 2 were commissioned and funded by Gilead Sciences and are under the copyright of Gilead Sciences, Inc. All rights reserved. Videos are used under licence from Gilead Sciences, who have had no input into the content of this research article.

EB received funds for speaking at symposia organised on behalf of Gilead. DT has received funds for consultation from Owlstone Medical in the last 5 years.

## Supporting information



Supporting Information

Supporting Information

Supporting Information

Supporting Information
