# Supplementary figures and images for: Microfluidic live‐cell imaging of Aspergillus fumigatus and Candida albicans hyphal growth treated with AmBisome and Caspofungin
Source: J Microsc. 2025 Dec 18;301(2):241–54. doi: 10.1111/jmi.70053 (PMC12884456; doi:10.1111/jmi.70053)

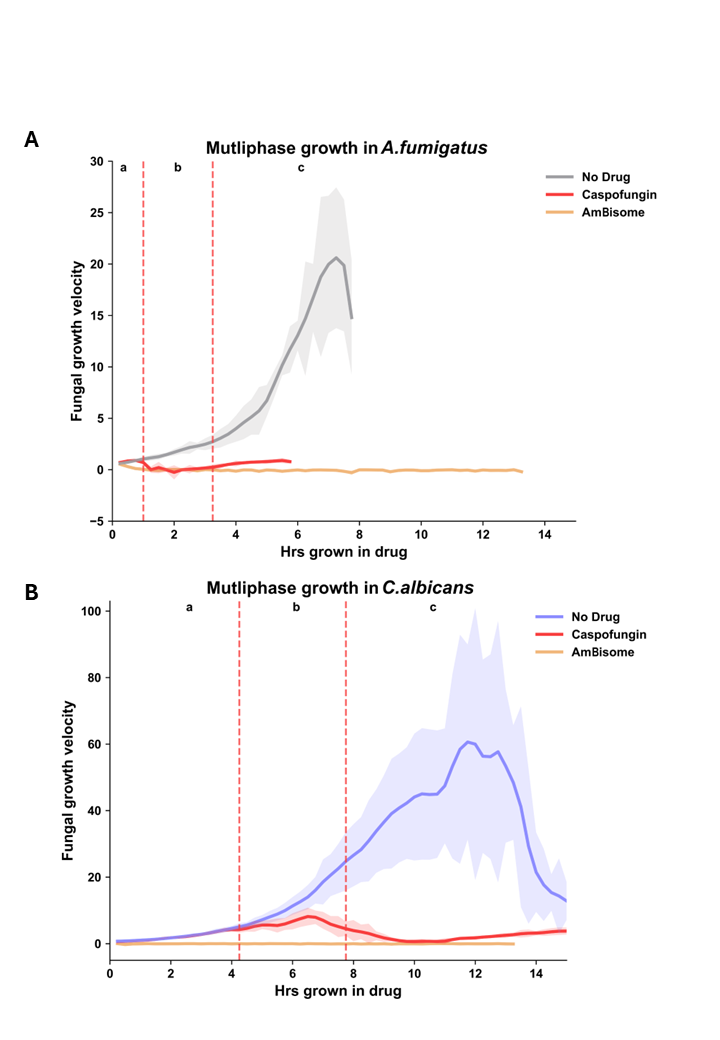

Supplement: Supplementary file 1 — Supporting Information [file JMI-301-241-s004.tif]

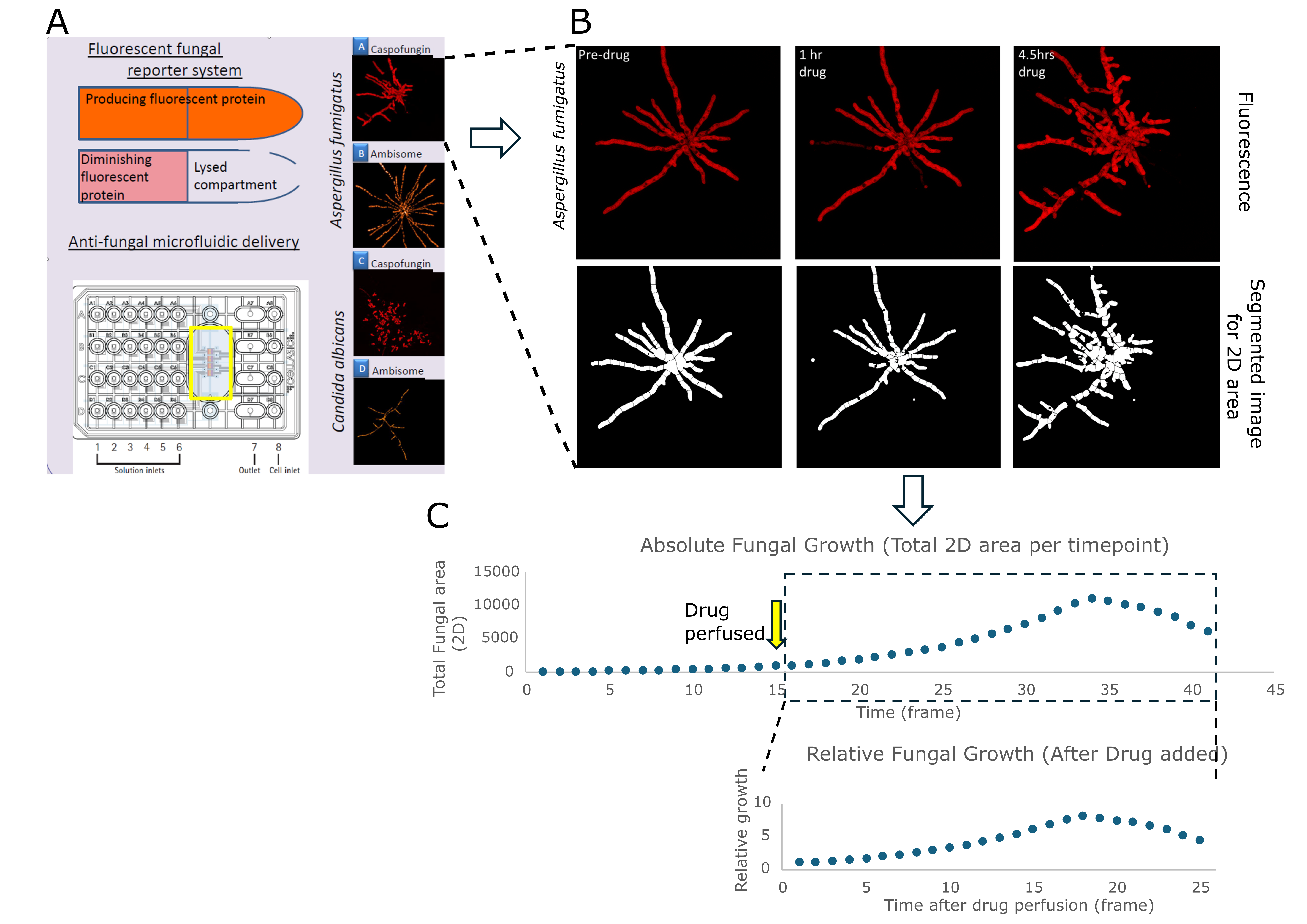

Supplement: Supplementary file 2 — Supporting Information [file JMI-301-241-s001.png]
